# Preperitoneal extended totally extraperitoneal (PeTEP) repair for ventral hernia: A systematic review and meta-analysis

**DOI:** 10.1007/s10029-026-03736-1

**Published:** 2026-06-02

**Authors:** Ana Luíza Rocha Soares Menegat, Brenda Luana Rocha Soares Menegat, Rafaela Duarte Vergna, Bernardo Wagner, Josélio Rodrigues de Oliveira Filho, Sergio Mazzola Poli de Figueiredo, Victor Kenzo Ivano

**Affiliations:** 1 University of Caxias Do Sul, 1130, Francisco Getúlio Vargas Street, Caxias Do Sul, 95070-560 Brazil; 2 State University of Rio Grande Do Norte, Natal, Brazil; 3 Escola de Medicina Souza Marques, Rio de Janeiro, Brazil; 4 Department of Pediatric Surgery, Massachusetts General Hospital, Harvard Medical School, Boston, USA; 5 Department of Surgery, University of North Carolina, Chapel Hill, USA; 6 Universidade Estadual de Campinas, Campinas, Brazil

**Keywords:** PeTEP, Preperitoneal Extended Totally Extraperitoneal, Ventral hernia, Meta-Analysis

## Abstract

**Purpose:**

Ventral hernias are common abdominal wall defects associated with substantial healthcare burden and impaired quality of life. Minimally invasive extraperitoneal techniques have evolved to optimize midline reconstruction while avoiding intraperitoneal mesh placement, especially in the setting of diastasis recti. Preperitoneal Extended Totally Extraperitoneal (PeTEP) repair is a recently introduced approach, but evidence regarding its safety and effectiveness remains limited. Therefore, we performed a systematic review and meta-analysis to synthesize the available evidence.

**Methods:**

A systematic search of PubMed, Embase, and the Cochrane Library was conducted to identify studies evaluating PeTEP repair for ventral hernia with or without rectus diastasis. Meta-analytical pooling of outcomes was performed using a random-effects model. All statistical analyses were conducted using R software (version 4.4.1).

**Results:**

Four studies were included, comprising 99 patients, with a mean age of 51.53 years and a mean body mass index of 29.55 kg/m^2^. The pooled mean operative time was 129.48 min (95% CI 74.96 to 184.00). Hematoma occurred in 10.61% of patients (95% CI 5.14 to 20.62), and overall postoperative complications in 21.21% (95% CI 12.98 to 32.69), with no intervention required in either case. Bulging was observed in 4.88% of cases (95% CI 1.22 to 17.52). No recurrences were reported across studies, with follow-up ranging from 1 to 12 months.

**Conclusion:**

Current evidence suggests that PeTEP may be a feasible minimally invasive extraperitoneal approach for ventral hernia repair. However, further comparative studies with longer follow-up are needed.

**Supplementary Information:**

The online version contains supplementary material available at 10.1007/s10029-026-03736-1.

## Introduction

Ventral hernias represent a frequent indication for surgery, with approximately 611,000 ventral hernia repairs performed annually in the United States, accounting for an estimated annual healthcare expenditure of $9.7 billion [1]. Contemporary international guidelines describe a broad spectrum of operative strategies but in recent years minimally invasive surgery and extraperitoneal techniques are increasingly utilized [2].

Among these extraperitoneal strategies, Preperitoneal Extended Totally Extraperitoneal (PeTEP) repair is a recently developed minimally invasive technique designed to address ventral hernia, with or without associated rectus diastasis, by allowing midline reconstruction and mesh placement in the preperitoneal plane while avoiding intraperitoneal access [3, 4]. Conceptually derived from extended totally extraperitoneal (eTEP) approaches, PeTEP provides wide bilateral access to the preperitoneal space, facilitates posterior plication of the rectus muscles, and enables adequate mesh overlap with limited or no fixation [5, 6]. Early case series and technical reports, including laparoscopic and robotic variants, suggest that PeTEP is feasible and safe, with promising early outcomes regarding recurrence and postoperative recovery.

Despite increasing adoption, the evidence supporting PeTEP remains limited. Published data consist mainly of small retrospective series and technical descriptions, with marked heterogeneity in patient selection, operative technique, and outcome reporting. Importantly, no randomized trials or large prospective comparative studies have evaluated PeTEP against established techniques for ventral hernia repair. As a result, the true magnitude, consistency, and reproducibility of PeTEP outcomes remain uncertain [7].

In light of the novelty of the technique and the absence of comparative trials, we performed a systematic review and meta-analysis to synthesize the currently available evidence on PeTEP repair in patients with ventral hernia with or without rectus diastasis and to provide a consolidated overview of reported clinical experience.

## Methods

### Protocol and registration

This systematic review and meta-analysis was conducted in accordance with the Cochrane Handbook for Systematic Reviews of Interventions. Reporting followed the Preferred Reporting Items for Systematic Reviews and Meta-Analyses (PRISMA) guidelines, and the completed PRISMA checklists are provided in Supplementary Tables 1 and 2 [8, 9]. The study protocol was prospectively registered in the International Prospective Register of Systematic Reviews (PROSPERO; registration number: CRD420261333162).

### Eligibility criteria

Studies were considered eligible if they met the following criteria: (1) inclusion of adult patients (≥ 18 years) presenting with ventral hernia (including umbilical, supraumbilical, paraumbilical, or epigastric), with or without rectus diastasis; (2) undergoing PeTEP repair; (3) prospective or retrospective single-arm observational studies, including structured case series with a minimum of 15 patients; and (4) reporting at least one outcome of interest relevant to this review.

Studies were excluded if they met any of the following criteria: (1) narrative reviews, systematic reviews, or meta-analyses; (2) case reports; (3) clinical practice guidelines, consensus statements, editorials, or correspondence; (4) conference abstracts lacking sufficient extractable data; (5) study protocols without published results; or (6) studies with overlapping patient populations. In cases of potential overlap, only one study was included to avoid duplication of data, and the primary or earliest published report was retained.

### Search strategy

A comprehensive literature search was performed in PubMed, Embase, and the Cochrane Central Register of Controlled Trials (CENTRAL) from database inception to November 2025. The search strategy combined free-text terms related to ventral hernia (e.g., “ventral hernia”, “umbilical hernia”, “epigastric hernia”, “paraumbilical hernia”, “supraumbilical hernia”) with terms describing pre-aponeurotic and preperitoneal endoscopic repair techniques, specifically “PeTEP” and “preperitoneal endoscopic totally extraperitoneal”, using Boolean operators (AND, OR).

All records identified through the electronic searches were imported into Rayyan (Rayyan Systems Inc., Qatar) [10], where duplicate entries were removed using automated and manual procedures. Two reviewers (A.L.R.S.M. and B.L.R.S.M.) independently screened titles and abstracts, followed by full-text evaluation of studies deemed potentially eligible. Discrepancies were resolved through consensus.

### Data extraction

Data extraction was performed independently by two reviewers (A.L.R.S.M. and B.L.R.S.M.) using a predefined and standardized data collection template. For each included study, the following variables were recorded: study characteristics (first author and year of publication), methodological design, length of follow-up, country where the study was conducted, sample size, sex distribution, mean or median age of participants, defect dimensions, hernia subtype, anatomical site of the hernia defect, body mass index (BMI), and surgical approach.

### Endpoints

The outcomes evaluated in this study encompassed both intraoperative and postoperative measures. The primary intraoperative outcome was operative time. Postoperative outcomes included hematoma formation, postoperative bulging, length of hospital stay, and overall postoperative complications, defined as the occurrence of seroma, hematoma, ileus, wound dehiscence, renal insufficiency, or bulging.

### Statistical analysis

All quantitative syntheses were conducted using a single-arm meta-analytic framework, considering the expected clinical and methodological variability among the included studies. For binary outcomes, pooled proportions were calculated using a generalized linear mixed-effects model (GLMM) with a logit link function (PLOGIT). Between-study variance (τ^2^) was estimated using the maximum likelihood (ML) approach. For continuous outcomes, pooled effects were synthesized as raw means (MRAW), with between-study variance estimated via the restricted maximum likelihood (REML) method [8].

The strategy for confidence interval construction was prespecified and applied uniformly across outcome types. Wald-type confidence intervals were used when no between-study heterogeneity was detected (τ^2^ = 0). When heterogeneity was present (τ^2^ > 0) and at least two studies contributed to the pooled estimate, the Hartung–Knapp adjustment was employed to yield more conservative inference [11].

Statistical heterogeneity was evaluated using Cochran’s Q test and quantified with the I^2^ statistic. In accordance with the Cochrane Handbook, I^2^ values of 0–40% were interpreted as possibly unimportant heterogeneity, 30–60% as moderate heterogeneity, 50–90% as substantial heterogeneity, and 75–100% as considerable heterogeneity. Ninety-five percent prediction intervals were calculated to reflect the expected range of true effects in future populations [12].

All analyses were performed using R software (R Foundation for Statistical Computing). Data management and preprocessing were conducted with the *rstudioapi* and *readxl* packages, and meta-analyses were carried out using the *meta* package [13]. Statistical significance was defined as a two-sided p-value < 0.05.

### Quality assessment

The methodological quality of the included single-arm observational studies was assessed using the Joanna Briggs Institute (JBI) Critical Appraisal Checklist for Studies Reporting Prevalence Data [14]. This instrument evaluates nine methodological domains, with each item classified as yes, no, unclear, or not applicable.

Although the JBI framework allows for an overall judgment (include, exclude, or seek additional information), no study was excluded based on quality alone. Instead, item-level assessments were used to characterize potential sources of bias and to guide interpretation of the pooled findings.

### Sensitivity analysis

The robustness of the pooled results was evaluated through sensitivity analyses based on a leave-one-out strategy, whereby the meta-analysis was repeatedly recalculated after sequentially excluding each study. This approach was used to assess whether the overall estimates were unduly driven by any single study or influenced by specific contributors to between-study variability [15].

In addition, Baujat plots were generated to graphically illustrate the relative contribution of each study to overall heterogeneity and its influence on the combined effect size, facilitating the identification of studies with a disproportionate impact on the meta-analytic findings [16]. Sensitivity analyses were conducted only for outcomes exhibiting substantial heterogeneity (I^2^ > 50%) and for which at least three studies were available for pooling.

### Assessment of publication bias

Potential publication bias was evaluated by visual inspection of funnel plots constructed for the pooled outcomes, with asymmetry considered a possible indicator of small-study effects, selective reporting, or publication bias [17]. To complement this qualitative assessment, funnel plot asymmetry was further examined using Egger’s regression test, with statistically significant results interpreted as suggestive of small-study effects [18].

Both funnel plot analysis and Egger’s regression were performed only for outcomes informed by at least three studies. For outcomes based on a limited number of contributing studies, these methods were interpreted with caution, given their reduced power and reliability in small meta-analyses.

## Results

### Study selection and baseline characteristics

**Fig. 1:**
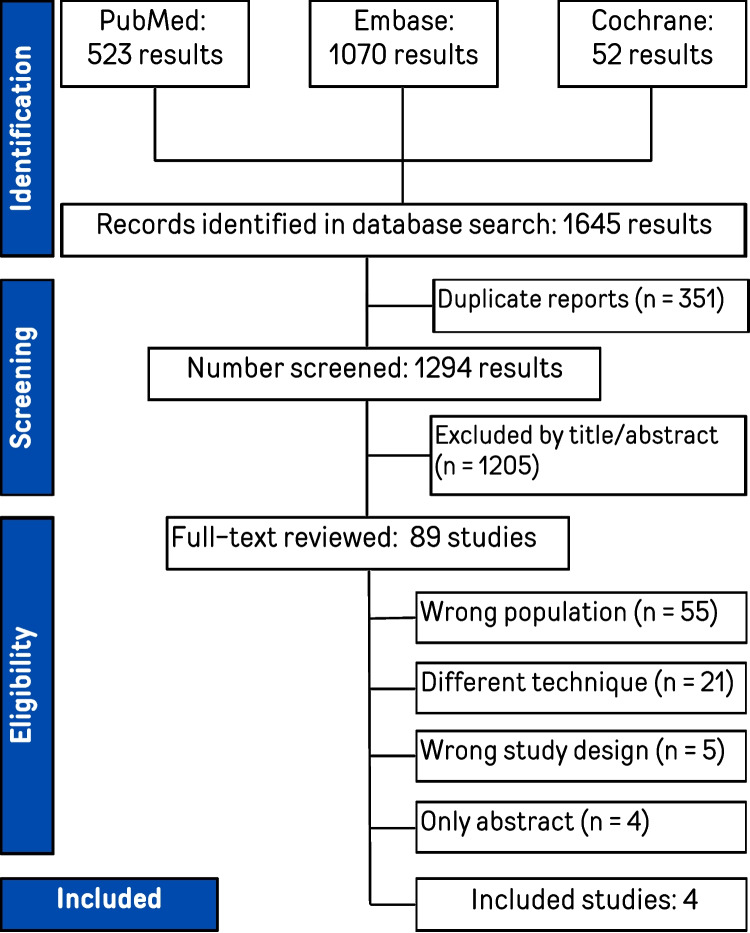
PRISMA flow diagram of study screening and selection

The literature search identified 1645 records across databases, including 523 from PubMed, 1070 from Embase, and 52 from Cochrane. After removal of 351 duplicate reports, 1294 records were screened by title and abstract, of which 1205 were excluded. A total of 89 full-text articles were assessed for eligibility. Of these, 85 studies were excluded for the following reasons: wrong population (n = 55), different surgical technique (n = 21), wrong study design (n = 5), and only abstract available (n = 4). Ultimately, 4 studies met the inclusion criteria and were included in the systematic review and meta-analysis (Fig. 1).

A total of 99 patients underwent PeTEP repair across the included studies. Rectus diastasis was present in 83 patients (83.83%). The study population comprised 37 females (37.37%) and 62 males (62.63%), with a mean age of 51.53 years. The mean hernia defect size was 2.95 cm. Regarding hernia type, 76 patients (76.77%) had primary hernias and 23 (23.23%) had incisional hernias. The mean BMI was 29.55 kg/m^2^, however, one study did not report BMI data. Follow-up across studies ranged from 1 to 12 months. Further details of baseline characteristics are provided in Table 1 [3, 6, 19, 20].

**Table 1:** Individual characteristics of the included studies. * Median; ** Mean. NR- not reported

***Study***	***Study Design***	***Follow-up (months)***	***Country***	***Rectus Diastasis n (%)***	***Number of Patients***	***Female/Male***	***Age*** ***(mean* ± *SD)***	***Defect Size*** ***(mean* ± *SD)***	***Hernia Type***	***Hernia Defect Location***	***BMI***	
												*Surgical Approach* *n (%)*
^**Alpuche 2024**^	Case series	12	Mexico	25 (76)	33	19/14	44.40 ± 10.10	Hernia 3.00**	Primary 33	Epigastric 1 Epigastric/Umbilical 8 Umbilical 24	NR	Laparoscopic 26 (78.79) Robotic 7 (21.21)
^**Arias−Espinosa 2024**^	Retrospective cohort	1*	USA, Mexico, and Brazil	25 (100)	25	2/23	53.74 ± 15.32	Hernia 3.35 ± 0.78 Diastasis 3.53 ± 1.02	Primary 21 Incisional 4	NR	30.86 ± 2.75	Robotic 25 (100)
^**Equisoain−Azcona 2025**^	Case series	2.9*	Spain	9 (52.90)	17	7/10	48.82 ± 12.43	Hernia 2.88 ± 1.62 Diastasis 4.25*	Primary 7 Incisional 10	Epigastric 1 Umbilical 7 Subxiphoidal/Epigastric 1 Subxiphoidal/Umbilical 1 Epigastric/Umbilical 6 Umbilical/Infraumbilical 1	30.25 ± 4.77	Laparoscopic 17 (100)
^**Munoz−Rodriguez 2025**^	Retrospective cohort	5.3**	Spain	24 (100)	24	9/15	59.17 ± 11.76	Hernia 2.58 ± 1.05 Diastasis 4.21 ± 0.83	Primary 15 Incisional 9	Epigastric 4 Umbilical 11 Midline incisional 7 Lateral incisional 2	27.89 ± 3.88	Laparoscopic 24 (100)

### Operative time

**Fig. 2:**
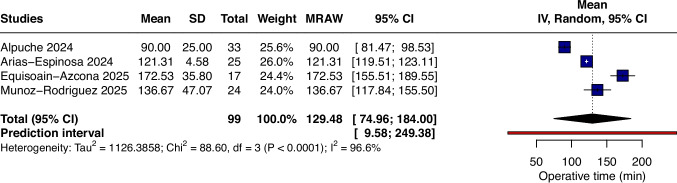
Forest plot of pooled mean operative time after PeTEP repair

Operative time following the PeTEP approach demonstrated a pooled mean of 129.48 min (95% CI 74.96 to 184.00; I^2^ = 96.60%; p < 0.0001; Fig. 2), with considerable heterogeneity across studies. The prediction interval was wide (PI 9.58 to 249.38), indicating that operative time in future studies may plausibly vary substantially.

### Hematoma

**Fig. 3:**
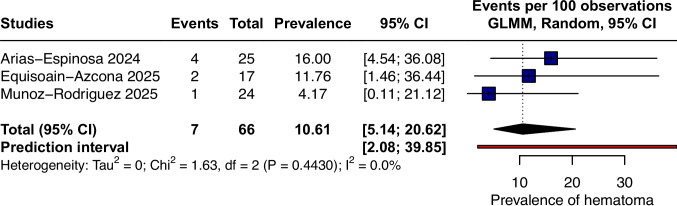
Forest plot of pooled prevalence of hematoma not requiring intervention after PeTEP repair

Hematoma following PeTEP repair demonstrated a pooled prevalence of 10.61% (95% CI 5.14 to 20.62; I^2^ = 0%; p = 0.4430; Fig. 3), indicating no observed statistical heterogeneity among the included studies. No cases required surgical or radiological intervention, as reported by the included studies. The prediction interval was wide (PI 2.08 to 39.85), indicating that the prevalence of hematoma in future studies may plausibly range across this interval.

### Postoperative complications

**Fig. 4:**
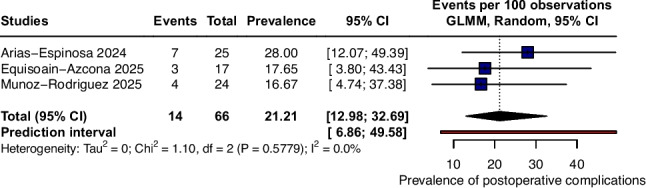
Forest plot of pooled prevalence of postoperative complications not requiring intervention after PeTEP repair

Postoperative complications following the PeTEP approach demonstrated a pooled prevalence of 21.21% (95% CI 12.98 to 32.69; I^2^ = 0%; p = 0.5779; Fig. 4), indicating no observed statistical heterogeneity among the included studies. Reported events were predominantly minor complications, and no cases required reoperation or invasive intervention. The prediction interval was wide (PI 6.86 to 49.58), indicating that the prevalence of postoperative complications in future studies may plausibly range across this interval.

### Bulging

Bulging following the PeTEP repair demonstrated a pooled prevalence of 4.88% (95% CI 1.22 to 17.52; I^2^ = 0%; p = 0.8025; Supplementary Fig. 1), with no observed heterogeneity. The prediction interval was wide (PI 0 to 99.81), indicating that the prevalence of bulging in future studies may plausibly range across this interval. Diagnostic criteria for bulging were not uniformly reported across studies: Equisoain-Azcona et al. did not specify how bulging was defined or diagnosed, whereas Munoz-Rodríguez et al. assessed patients clinically during follow-up and performed abdominal CT with Valsalva maneuver when bulging was suspected. 

### Length of hospital stay

Length of hospital stay following the PeTEP approach demonstrated a pooled mean of 0.69 days (95% CI − 6.49 to 7.87; I^2^ = 99.0%; p < 0.0001; Supplementary Fig. 2), with considerable heterogeneity. The prediction interval was wide (PI −11.70 to 13.08), indicating that length of hospital stay in future studies may plausibly vary markedly.

### Systematic review

#### Suture materials used for rectus diastasis repair

Across the included studies, repair of rectus diastasis during the PeTEP procedure was consistently performed using barbed sutures, with only minor variations in suture material, absorbability, caliber, and suturing technique. Alpuche et al. used a non-absorbable barbed suture (No. 1) for midline plication, whereas the remaining studies predominantly employed slowly absorbable barbed sutures in calibers ranging from USP 0 to USP 1, including V-Loc™ 180 in the robotic series by Arias-Espinosa et al. Similarly, Muñoz-Rodríguez et al. reported the use of caliber 0 (USP 0) barbed sutures for rectus diastasis closure [3, 6, 19]. Notably, Equisoain-Azcona et al. adopted a barbed inverted suture technique specifically for diastasis plication, representing a technical nuance rather than a fundamentally different approach [20]. Overall, these variations reflect expected differences in operative preference and device availability rather than substantive departures from a shared technical framework.

#### Mesh characteristics and fixation

Across the included studies, prosthetic reinforcement during PeTEP repair was performed using synthetic meshes, with some variability in mesh material, weight, and fixation strategy. Alpuche et al. used a middle-weight polypropylene mesh without penetrating fixation, suggesting glue fixation only if deemed necessary [3]. Arias-Espinosa et al. employed a medium-weight polypropylene or monofilament polyester mesh with large pores (25 × 12 cm), with additional fixation rarely required and limited to a few cephalic and caudal sutures when deemed necessary [19]. Muñoz-Rodríguez et al. used a medium-weight, wide-pore polypropylene mesh placed without any fixation [6]. Equisoain-Azcona et al. reported the use of a medium-weight polypropylene mesh (approximately 30 × 15 cm) placed without fixation, ensuring flat positioning against the muscular wall during progressive cavity deflation [20]. These differences reflect routine institutional preferences and device availability rather than fundamentally different operative concepts.

#### Patient-Reported and quality-of-Life–Related Outcomes

Across the included studies evaluating PeTEP repair and its laparoscopic or robotic variants, patient-centered outcomes suggest a favorable impact on postoperative quality of life, although none of the studies employed validated quality-of-life instruments. Collectively, the reports describe low postoperative pain, short hospital length of stay (often same-day discharge or within 24 h), and rapid functional recovery, consistent with the minimally invasive and extraperitoneal nature of the technique. Authors also emphasize preservation of the posterior rectus sheath and avoidance of the retromuscular plane, which may translate into improved abdominal wall function, reduced risk of chronic pain, and better cosmetic outcomes. Overall, these findings describe a postoperative profile that is compatible with good short-term patient-reported recovery, while underscoring the absence of standardized quality-of-life assessment.

#### Aesthetic and cosmetic aspects

Across the included studies, aesthetic aspects were discussed descriptively rather than assessed using standardized cosmetic or body image scales. Authors commonly highlighted the small size and limited number of skin incisions, as well as their strategic placement away from the midline, as factors contributing to favorable cosmetic appearance. The extraperitoneal approach and avoidance of large open incisions were also reported to minimize visible scarring and reduce abdominal wall contour irregularities. In cases with associated rectus diastasis, midline plication was described as restoring abdominal wall anatomy, which may contribute to improved anterior abdominal contour. Overall, the available evidence characterizes aesthetic outcomes as acceptable to favorable, while underscoring the lack of objective or patient-reported cosmetic outcome measures.

#### Recurrence assessment and detection methods

Recurrence reporting varied across the included studies in terms of surveillance strategy and duration of follow-up, limiting direct comparability. All four studies reported no recurrences. However, these findings should be interpreted with caution, as the follow-up duration is insufficient to reliably assess recurrence rates in ventral hernia repair. Muñoz-Rodríguez et al. reported a mean follow-up of 5.3 months, Equisoain-Azcona et al. a median follow-up of 87 days, and Arias-Espinosa et al. a median follow-up of 30 days [6, 19, 20]. In the robotic series by Arias-Espinosa et al., recurrence was assessed by clinical evaluation, patient self-reporting and/or postoperative imaging [19]. Alpuche et al. reported that, in case of suspected recurrence, clinical evaluation and CT were always performed, although imaging was not used routinely in asymptomatic patients [3]. The remaining studies relied primarily on clinical follow-up, and the use of systematic imaging surveillance was not clearly described. These features indicate that the apparent absence of recurrence across studies should be interpreted in the context of short observation windows and variable detection methods.

#### Postoperative pain and analgesic requirements

Postoperative pain outcomes were heterogeneously reported across the included studies, but the available evidence suggests a generally favorable pain profile following PeTEP repair. Muñoz-Rodríguez et al. reported that no patients required opioid analgesia beyond 48 h postoperatively, and no cases of chronic pain were documented during follow-up [6]. Alpuche et al. reported that no narcotic analgesics were used during the hospital stay or at follow-up, although chronic pain was not specifically assessed or reported [3]. Equisoain-Azcona et al. reported that only three patients required a prolonged hospital stay of two days due to postoperative pain management, with no further long-term pain outcomes described [20]. Importantly, none of the studies applied uniform pain measurement tools or consistent follow-up time points, and no study was designed to compare pain outcomes with alternative surgical techniques.

### Quality assessment

Methodological quality and risk of bias of the included studies are presented in Supplementary Table 3. The studies were appraised using the JBI Critical Appraisal Checklist for Studies Reporting Prevalence Data. Overall, the included studies demonstrated acceptable methodological quality, with most appraisal items rated as “Yes”. A recurrent methodological limitation concerned sample size adequacy, which was consistently rated as “No”, reflecting the small and retrospective nature of the available evidence. The appraisal item related to response rate was judged as not applicable across all studies. Based on the overall JBI assessment, all studies were considered methodologically suitable for inclusion in the quantitative synthesis, and none were excluded based on methodological quality.

### Sensitivity analysis

Sensitivity analysis for operative time was conducted using leave-one-out and Baujat plots. In the leave-one-out analysis, heterogeneity decreased only slightly with omission of each individual study, however, exclusion of Alpuche et al. resulted in the greatest reduction in heterogeneity, with I^2^ decreasing to 94.60% (Supplementary Fig. 3) [3]. Consistently, the Baujat plot identified the same study, Alpuche et al., as the main contributor to overall heterogeneity (Supplementary Fig. 4) [3]. Despite this influence, the pooled effect remained stable, supporting the robustness of the findings for operative time.

### Publication bias

Publication bias was assessed for operative time, hematoma, and postoperative complications (Supplementary Figs. 5–7). Visual inspection of the funnel plots showed a symmetric distribution of studies for all outcomes. Consistently, Egger’s test did not indicate significant small-study effects for operative time (p = 0.9065), hematoma (p = 0.1557) or postoperative complications (p = 0.2205), suggesting no evidence of publication bias for these endpoints.

## Discussion

In this systematic review and meta-analysis including four observational studies and 99 patients undergoing PeTEP repair for ventral hernia with or without rectus diastasis, we evaluated perioperative outcomes and postoperative morbidity. Overall, PeTEP repair was associated with acceptable operative times, low rates of clinically significant complications, and a short length of hospital stay. Postoperative morbidity was generally low, with most complications being minor and not requiring intervention, and postoperative bulging was infrequent. Taken together, these findings provide a general overview of the current evidence regarding perioperative outcomes following PeTEP repair.

The PeTEP technique was first described by Valenzuela Alpuche et al. as a minimally invasive endoscopic extraperitoneal approach for midline abdominal wall reconstruction, representing a relatively recent addition to the surgical armamentarium for ventral hernia repair and rectus diastasis [3]. International guidelines broadly converge in recommending mesh-based repair as the preferred treatment for most ventral and incisional hernias, although nuances exist regarding approach and mesh position. The updated International Endohernia Society (IEHS) guidelines recommend mesh reinforcement in patients with concomitant rectus diastasis and acknowledge that novel minimally invasive extraperitoneal techniques, including MILOS, eMILOS, and eTEP, may be offered, although long-term outcome data remain limited [2]. As a recently developed minimally invasive extraperitoneal approach, PeTEP remains outside the scope of current guideline statements, highlighting the need for synthesis to clarify its safety and effectiveness.

Minimally invasive extraperitoneal approaches for ventral hernia repair encompass both preperitoneal and retromuscular techniques, which differ primarily in their dominant dissection plane and extent of posterior myofascial manipulation [21]. These approaches may also address concomitant rectus diastasis when present. Retromuscular approaches, including open and minimally invasive Rives–Stoppa–based techniques and posterior component separation variants, achieve defect closure and diastasis correction through posterior rectus sheath division, providing robust medialization and mesh reinforcement [22, 23]. Procedure-specific complications reported in retromuscular techniques, including eTEP, include injury to the posterior rectus sheath, linea alba disruption, and injury to segmental neurovascular bundles [24]. In addition, postoperative abdominal wall bulging has been described following retromuscular repairs, particularly when posterior sheath division or releasing incisions are performed [25]. In contrast, the PeTEP approach achieves defect closure and mesh reinforcement through a preperitoneal plane, without routine division of the posterior rectus sheath [4]. By avoiding disruption of this structure, the PeTEP approach provides direct access to the preperitoneal plane and may reduce manipulation of the neurovascular bundles supplying the rectus abdominis [19]. Currently, direct comparative studies between PeTEP and retromuscular techniques for ventral hernia and rectus diastasis repair are lacking, with available evidence derived from non-comparative series.

Minimally invasive approaches for the repair of ventral hernia with rectus diastasis have been increasingly adopted, with several techniques reporting favorable perioperative outcomes. In comparison with our findings, operative time for PeTEP appears shorter than that reported for other retromuscular approaches, including eTEP (median 164.50 min) and EMILOS (mean 157.60 min), although shorter operative times have been described with SCOLA (93.50 min) [22, 26, 27]. Length of hospital stay following PeTEP also appears reduced compared to eTEP (3 days) and EMILOS (3.20 days) series [22, 26].

Regarding postoperative morbidity, PeTEP demonstrated rates of hematoma and overall postoperative complications that fall within the range reported in other minimally invasive approaches. For instance, hematoma rates in eTEP series have been reported as low as 2.32%, while overall postoperative complications in SCOLA cohorts may reach up to 31.20% [27, 28]. Similarly, bulging rates appear lower in PeTEP compared to non-stressed bulging reported in eTEP series (11.10%) [29]. In this context, indirect comparisons with other minimally invasive approaches may help to contextualize the observed outcomes.

Some limitations should be acknowledged. First, most available evidence derives from observational studies without comparative groups, which is largely attributable to the relatively recent introduction of this technique. As a result, direct comparisons between surgical approaches were not feasible. Additionally, the overall sample size across studies was relatively small, which may limit the generalizability of the findings. Furthermore, for some outcomes, quantitative analyses were based on only two studies, limiting statistical power and precluding robust assessment of between-study heterogeneity or small-study effects. The wide confidence intervals observed for some outcomes further reflect the limited sample size and heterogeneity among included studies. In addition, the short follow-up duration across studies limits the ability to adequately assess longer-term outcomes.

## Conclusion

This systematic review and meta-analysis synthesizes the currently available evidence on perioperative outcomes following PeTEP for ventral hernia repair with or without rectus diastasis. The findings suggest that this technique may be feasible within the spectrum of minimally invasive approaches. However, these results should be interpreted with caution, as the current evidence is limited to small, non-comparative observational studies with heterogeneous populations and short follow-up. Further well-designed comparative studies are required to better define the role of PeTEP and to clarify its long-term effectiveness and durability.

## Supplementary Information

Below is the link to the electronic supplementary material.

Supplementary file1 (DOCX 5331 KB)

## Data Availability

The data supporting the findings of this study were obtained from previously published studies that are publicly available. All data extracted and analyzed are included within the article and its supplementary materials.
